# A case of frequent of prerenal acute kidney injury attacks: importance of recognizing systemic capillary leak syndrome: a case report

**DOI:** 10.1177/03000605241301863

**Published:** 2024-11-26

**Authors:** Reina Miyazawa, Hironori Nakamura, Michiko Kumagai, Mariko Anayama, Yasushi Makino, Marina Nishikawa, Koji Hashimoto, Yuji Kamijo

**Affiliations:** 1 Department of Nephrology, 38382 Shinonoi General Hospital, Nagano, Japan; 2 Department of Nephrology, Shinshu University Hospital, Matsumoto, Japan

**Keywords:** Case report, hypotension, haemoconcentration, hypoalbuminaemia

## Abstract

Systemic capillary leak syndrome (SCLS) is a rare and life-threatening disorder. A man in his 60s presented for emergency care because of fatigue, decreased urine output and difficulty in moving his body. On admission, he was conscious, afebrile and had relative hypotension. Blood tests and urinary analysis revealed the following: white blood cell count, 19 500/μl; haematocrit, 64.5%; creatinine, 2.16 mg/dl; albumin, 3.3 g/dl; and 0.2% for fractional excretion of sodium. The patient was diagnosed with prerenal acute kidney injury (AKI) and was treated with intravenous fluid administration of more than 2 l/day. His kidney function gradually recovered after 4 days and creatinine decreased (1.15 mg/dl). However, he developed two more attacks of prerenal AKI during hospitalization, one of which needed intensive care unit management. Specific findings of hypotension, haemoconcentration, and hypoalbuminaemia were observed during all AKI attacks. Finally, he was diagnosed as idiopathic SCLS and was treated with intravenous immunoglobulin. SCLS might remain undiagnosed because of its rarity, but it can rapidly progress and lead to severe complications in absence of treatment. Clinicians need to consider this disease as a differential diagnosis when encountering patients who present with frequent prerenal AKI attacks accompanied by hypotension, haemoconcentration and hypoalbuminaemia.

## Introduction

Systemic capillary leak syndrome (SCLS) is a rare and life-threatening disorder that was first described in 1960 as cyclical oedema and shock secondary to increased capillary permeability.^1^ SCLS is characterized by recurrent episodes of hypotension, haemoconcentration and hypoalbuminaemia as a consequence of leakage of plasma and proteins into the extravascular space. Since 1960, only approximately 260 cases have been reported,^2^ and because of its rarity and high mortality,^3^ SCLS might be easily undiagnosed. This current report describes a case of frequent attacks of prerenal acute kidney injury (AKI), which was finally diagnosed as idiopathic SCLS on the fourth recurrent episode of AKI.

## Case report

**Figure 1.**
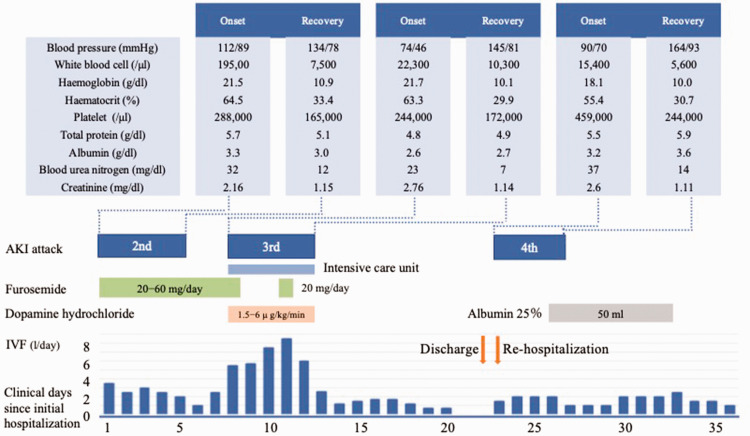
The three acute kidney injury (AKI) attacks and the respective laboratory data at the onset and recovery phase of each AKI attack for a male patient in his 60s who presented for emergency care because of fatigue, decreased urine output and difficulty in moving his body: data are shown in relation to the timeline, treatment and point of care during the clinical course of the patient before the transfer to a tertiary medical institution. IVF, intravenous fluid. The colour version of this figure is available at: http://imr.sagepub.com.

In August 2023, a man in his 60s was transported to the Emergency Department, Shinonoi General Hospital, Nagano, Japan for emergency care because of fatigue, decreased urine output and difficulty in moving his body. He did not have any signs of prior infection. He had been on medications for hypertension and hyperuricaemia. Two months prior, he was treated at another hospital because of severe dehydration and AKI for 12 days (first attack). Bone marrow tests were performed at the previous hospital and the patient had been followed-up without treatment. On the current admission, he was conscious and his body temperature was 37.2°C, his heart rate was 81 beats/min (bpm) and his blood pressure was 102/83 mmHg. He was anuric but showed no signs of extremity oedema. Intravascular fluid volume was evaluated by echocardiography, which showed a collapsed left atrium, nondilated inferior vena cava and respiratory collapse. Blood tests revealed the following: white blood cell (WBC) count, 19 500/μl; haemoglobin, 21.5 g/dl; haematocrit, 64.5%; creatinine (Cr), 2.16 mg/dl; albumin (Alb), 3.3 g/dl; and fractional excretion of sodium, 0.2% (Table 1). Based on these findings, the patient was diagnosed as prerenal AKI and was treated with more than 2 l/day of intravenous fluid (IVF). Considering the risk of heart failure, furosemide was also used. His kidney function gradually recovered after 4 days, with Cr at 1.15 mg/dl; and this AKI was defined as the second attack (Figure 1). After gradual reduction of the IVF volume to 1 l/day, he suddenly manifested with malaise and anuria, followed by shock. He was transferred to the intensive care unit on day 8. Blood tests at this time revealed the following: WBC count, 22 300/μl; haemoglobin, 21.7 g/dl; haematocrit, 63.3%; Cr, 2.76 mg/dl; and albumin, 2.6 g/dl. He was treated with massive IVF infusions and 1.5–6.0 µg/kg per min of dopamine hydrochloride. During this third attack, he developed pleural effusion and extremity oedema, which were treated with 20 mg furosemide intravenous once a day for 1 day. Echocardiogram showed preserved ejection fraction and no wall motion abnormality. Four days after the third attack, his kidney function gradually recovered to a Cr of 1.14 mg/dl; and this AKI was defined as the third attack (Figure 1). Thereafter, the IVF infusion was gradually decreased in volume and discontinued on day 21. He was discharged without symptoms, but the aetiology of prerenal AKI remained unknown. The next day, he was rehospitalized because of faintness and decreased urine output. His blood pressure was 90/70 mmHg and his heart rate was 88 bpm. Blood tests revealed the following: WBC count, 15 400/μl; haemoglobin, 18.1 g/dl; haematocrit, 55.4%; Cr, 2.6 mg/dl; and albumin, 3.2 g/dl; and this AKI was defined as the fourth attack (Figure 1). These findings of hypotension, haemoconcentration and hypoalbuminaemia were almost the same pattern as those seen in the previous two attacks. Although proteinuria and haematuria were detected on admission, these did not persist during his hospital stay. Myoglobinuria was not checked. Immunoglobulin (Ig)G kappa M protein was detected in the serum, but kappa/lambda ratio was not checked. Computed tomography scan and urinalysis ruled out postrenal or intrarenal AKI. After excluding polycythaemia vera, hereditary angioedema, medications and sepsis as the cause of prerenal AKI,^4,5^ and based on the specific clinical features of hypotension, haemoconcentration and hypoalbuminaemia during the acute onset of prerenal AKI and the response to IVF administration, idiopathic SCLS was finally diagnosed on day 27. Fortunately, he did not have severe organ abnormalities or vascular embolism during the course of his AKI attacks. Given the inadequate supply of immunoglobulin in our medical area, the patient was transferred to a tertiary medical institution in Matsumoto, Japan where he suffered another AKI attack. This led to the need for the patient to receive 70 g (1 g/kg) of intravenous immunoglobulin (IVIG) on days 41, 42 and 80; followed by 50 g of IVIG on days 154 and 209. The levels of serum Cr, Alb and IgG were 1.08 mg/dl, 4.5 g/dl and 1,633 mg/dl, respectively, on day 209. No recurrence of AKI has been observed for 8 months after the initiation of IVIG. Written informed consent was obtained from the patient for publication of this case report. The reporting of this study conforms to CARE guidelines.^6^ All patient details have been deidentified.

**Table 1.** Laboratory data on admission for a male patient in his 60s who presented for emergency care because of fatigue, decreased urine output and difficulty in moving his body.

White blood cell count, /μl	19 500	Total bilirubin, mg/dl	0.8	IgG, mg/dl	730
Neutrophil, %	83.5	Aspartate aminotransferase, IU/l	36	IgA, mg/dl	67
Eosinophil, %	0.2	Alanine aminotransferase, IU/l	24	IgM, mg/dl	37
Basophil, %	0.5	γ-glutamyl transpeptidase, IU/l	41		
Monocyte, %	3.4	Lactate dehydrogenase, IU/l	360	pH	5.0
Lymphocyte, %	12.4	Creatine phosphokinase, U/l	85	Specific gravity	1.011
Red blood cell count, /μl	6 620 000	Blood urea nitrogen, mg/dl	32	Protein	1+
Haemoglobin, g/dl	21.5	Creatinine, mg/dl	2.16	Glucose	–
Haematocrit, %	64.5	Sodium, mEq/l	136	Occult blood	3+
Platelet count, /μl	288 000	Potassium, mEq/l	6.6	Red blood cells, /HPF	20–29
		Chloride, mEq/l	104	White blood cells, /HPF	5–9
Total protein, g/dl	5.7	Calcium, mg/dl	8.7	NAG, U/l	15
Albumin, g/dl	3.3	C-reactive protein, mg/dl	0.05	FENa, %	0.2

Ig, immunoglobulin; HPF, high power field; NAG, N-acetyl-beta-D-glucosaminidase; FENa, fractional excretion of sodium.

## Discussion

A previous study reported that the median time from symptom onset to SCLS diagnosis was 1.1 years (interquartile range, 0.5–4.1 years);^5^ but the reported frequency of SCLS attacks varies considerably among patients, from one every 10 years to every 3–5 days^7^ or a median annual frequency of 1.23 (range, 0.13–21.18) per patient.^3^ It is highly likely that its rarity and the long interval between attacks contribute, at least, to the delay or difficulty in the diagnosis of SCLS. In the present case, it took approximately 4 weeks to diagnose the patient as SCLS. The reason for this relatively early diagnosis was that the disease manifested three times within a 4-week period and occurred during hospitalization.

Systemic capillary leak syndrome is a diagnosis of exclusion and has no established clinical definition or accepted diagnostic criteria.^7,8^ In clinical practice, a triad of Hs in the absence of other secondary causes are important to aid diagnosis: (i) hypotension, typically systolic blood pressure <90 mmHg; (ii) haemoconcentration (haematocrit > 49% to 50% in men and 43% to 45% in women); and (iii) hypoalbuminaemia (<3.0 g/dl).^9^

In the prodromal phase of SCLS, patients may develop decreased general condition, such as malaise, fatigue, generalized weakness or gastrointestinal symptoms. This is followed by the leak phase, which is characterized by rapid development of shock and anasarca secondary to plasma extravasation and usually lasts for several days. The post leak phase usually begins 48 h to 1 week after the onset of shock and is characterized by fluid mobilization from the peripheral tissues into the intravascular space, followed by normalization of blood pressure and diuresis.^9^ In the present case, the patient manifested with malaise, faintness and decreased urine output at the onset. The laboratory data shown as ‘onset’ and ‘recovery phase’ in the Figure 1 during AKI attacks could approximately correspond with the leak and post leak phases. AKI is a common manifestation of SCLS.^5^ The most common causes of AKI are prerenal secondary to intravascular volume depletion,^4,5^ but rhabdomyolysis reportedly developed in approximately one-third of patients.^5^ In this current case, the patient had AKI during each SCLS attack. Although the highest serum Cr levels were in the range of 2.16–2.76 mg/dl, his renal function fully recovered to the recovery phase levels after IVF administration during the three attacks, suggesting that intravascular fluid depletion secondary to plasma and protein loss contributed to AKI. In contrast, sepsis has a different pathophysiological mechanism of prerenal AKI and is characterized by the presence of infection. In particular, the cause of prerenal AKI in sepsis is reduced systemic vascular resistance or renal vasoconstriction in the early stage, whereas that for SCLS is intravascular volume depletion.^4^

Several studies have reported that monoclonal gammopathy of undetermined significance (MGUS) and multiple myeloma were associated with SCLS.^10^ A high level of monoclonal component (≥5 g/l) at diagnosis reportedly increased the susceptibility to severe relapse but was not associated with prognosis.^11^ This finding emphasized the role of monoclonal gammopathy or the plasma cell clone in the pathophysiology of SCLS.^11^ In the present case, the high monoclonal component level of 7.3 g/l might have been associated with the frequent relapses of AKI. Notably, the patient will need to undergo regular monitoring for multiple myeloma, based on the recommendation of a previous case report on a patient who developed multiple myeloma after the diagnosis of SCLS.^12^

Intravenous immunoglobulin has emerged as the most commonly used and effective first-line prophylactic therapy for SCLS.^2,13,14^ A systematic review of 263 cases of idiopathic SCLS estimated a 10-year survival rate of 93.8% in those who received IVIG and 48.5% in those who did not receive IVIG.^2^ Based on its potential immunomodulatory and anticytokine properties, IVIG has been widely used for the treatment of autoimmune, MGUS-associated syndromes,^15^^,1^^6^ and systemic inflammatory diseases. The mechanism of action of IVIG in patients with SCLS is not fully understood and complex, but it might involve the following: (i) neutralization of pathological antibodies; (ii) Fc receptor blockade; (iii) complement inhibition; (iv) immunoregulation of dendritic cells, B cells and T cells; and (v) modulation of apoptosis.^16^ Previous authors speculated that the antibodies in the IVIG preparations exert prophylactic effects in patients with SCLS through anti-idiotypic effects on the putative monoclonal antibody or by neutralizing proinflammatory cytokines.^9^ The present case was treated with IVIG for 8 months and no AKI recurrence was observed for at least 8 months after treatment. This was supported by a report that median attack frequency declined from 2.6 episodes/patient per year to 0 after commencement of IVIG prophylaxis.^9^ In the present case, no definitive answer is available for duration of IVIG administration or discontinuation of IVIG.

Systemic capillary leak syndrome might remain undiagnosed because of its rarity, but it can rapidly progress and lead to severe complications in the absence of treatment. Clinicians need to consider this disease as a differential diagnosis when encountering patients with frequent attacks of prerenal AKI accompanied by hypotension, haemoconcentration and hypoalbuminemia. Further accumulation of published cases is required to elucidate the mechanisms and pathogenesis of SCLS.
