# Corrigendum to “A case of frequent of prerenal acute kidney injury attacks: importance of recognizing systemic capillary leak syndrome: a case report”

**DOI:** 10.1177/03000605241311972

**Published:** 2024-12-31

Miyazawa R, Nakamura H, Kumagai M, et al. A case of frequent of prerenal acute kidney injury attacks: importance of recognizing systemic capillary leak syndrome: a case report. *J Int Med Res* 2024; 52(11). doi:10.1177/03000605241301863

There was an error in the article title. The correct title is ‘A case of frequent prerenal acute kidney injury attacks: importance of recognizing systemic capillary leak syndrome: a case report’.

Online version of the article has been corrected.

